# A comparative study of mesenchymal stem cell transplantation with its paracrine effect on control of hyperglycemia in type 1 diabetic rats

**DOI:** 10.1186/2251-6581-13-76

**Published:** 2014-08-11

**Authors:** Ehsan Aali, Solmaz Mirzamohammadi, Habib Ghaznavi, Zahra Madjd, Bagher Larijani, Samira Rayegan, Ali M Sharifi

**Affiliations:** Razi Drug Research Center and Department of Pharmacology, Iran University of Medical Sciences, Tehran, Iran; Department of Tissue Engineering and Cell Therapy, School of Advanced Technologies in Medicine, Iran University of Medical Sciences, Tehran, Iran; Endocrine and Metabolism Research Institute, Shariati Hospital, Tehran University of Medical Sciences, Tehran, Iran; Oncopathology Research Center and Department of Pathology, Iran University of Medical Sciences, Tehran, Iran

**Keywords:** Hyperglycemia, Mesenchymal stem cells, Diabetes mellitus

## Abstract

**Background:**

Many studies suggested mesenchymal stem cells (MSCs) transplantation as a new approach to control hyperglycemia in type 1 diabetes mellitus through differentiation mechanism. In contrary others believed that therapeutic properties of MSCs is depends on paracrine mechanisms even if they were not engrafted. This study aimed to compare these two approaches in control of hyperglycemia in STZ-induced diabetic rats.

**Methods:**

Animals were divided into five groups: normal; diabetic control; diabetic received MSCs; diabetic received supernatant of MSCs; diabetic received co-administration of MSCs with supernatant. Blood glucose, insulin levels and body weight of animals were monitored during experiment. Immunohistochemical and immunofluorescence analysis were performed to monitor functionality and migration of labeled-MSCs to pancreas.

**Results:**

First administration of MSCs within the first 3 weeks could not reduce blood glucose, but second administration significantly reduced blood glucose after week four compared to diabetic controls. Daily injection of supernatant could not reduce blood glucose as efficient as MSCs. Interestingly; Co-administration of MSCs with supernatant significantly reduced blood glucose more than other treated groups. Insulin levels and body weight were significantly increased in MSCs + supernatant-treated animals compared to other groups. Immunohistological analysis showed an increase in number and size of islets per section respectively in supernatant, MSCs and MSCs + supernatant-treated groups.

**Conclusion:**

Present study exhibited that repeated-injection of MSCs reduced blood glucose and increased serum insulin levels in recipient rats. Injection of supernatant could not reverse hyperglycemia as efficient as MSCs. Interestingly; co-administration of MSCs with supernatant could reverse hyperglycemia more than either group alone.

## Background

Type 1 diabetes mellitus (T1DM) is an autoimmune disease in which β-cells are completely destroyed, resulting in metabolic dysfunction as a consequence of insufficient circulating levels of insulin [1, 2]. Insulin therapy, as a principal method of treatment, not only has relative life-threatening complications but also do not able to mimic exactly the physiology of insulin secretion in the body [3]. The alternative method to treat diabetes are transplantation of whole pancreas and/or pancreatic islets cells [4]. Pancreatic islets transplantation has major limitations, including inadequate organ donor and also many complications of rejection reaction when xenogenic islet cells have been used [5]. Hence, much effort has been made to generate β cell mass either by stimulating endogenous regeneration of islets or in vitro differentiation of islet-like cells [6–8]. Many studies have revealed that stem/progenitor cells, can promote injured tissue repair. Since MSCs could easily be isolated from bone marrow and differentiated into a variety of cell types, being known as the most commonly used stem cells in tissue engineering and regenerative medicine [9, 10]. The ability of mesenchymal stem cells particularly bone marrow derived (BM-MSCs) to differentiate into many cell types, as well as their high expansion potential ex vivo, makes them an attractive therapeutic tool for cell transplantation and tissue engineering [11–14]. Several evidences suggested that administration of MSCs have highly potential to recover endogenous β cells in diabetes mellitus due to their ability to differentiate into many cells and tissues under specific condition [15–17]. However, the precise function of BM-derived cells in β cell regeneration is controversial. Some studies believe that therapeutic properties and use of MSCs would be primarily based on their paracrine mechanisms by releasing trophic and immunomodulatory factors. It has been demonstrated that MSCs produce and release various growth factors and cytokines including anti-apoptotic, immunomodulatory, supportive, angiogenic, chemoattractic factors. These studies indicated that MSCs are promising tools for the treatment of different types of tissue damages, because they secrete a multitude of bioactive molecules that ultimately lead to reformation and regeneration of injured tissues [18–21]. Therefore, many studies shift their focus from differentiation to paracrine mechanism in which MSCs could be used as therapeutic tools, even if they do not engraft or differentiate into tissue-specific cell [19, 21, 22]. It has been shown that MSCs migrate and dock preferentially into the injured or damaged tissue sites promoting the survival of surrounding cells [21, 23, 24].

This study was designed to assess: [1] Beneficial effect of supernatant of MSCsin control of hyperglycemia in diabetic rats [2]. Comparative evaluation of MSCs transplantation with their supernatant administration in control of hyperglycemia in diabetic rats [3]. The beneficial effects of co-administration of MSCs with supernatant in control of T1DM.

## Material and methods

### Animals

Eight-weeks-old male Wistar rats with initial body weights of 200-250 g were used in this study. The rats were divided into five groups, normal control, diabetic control, MSCs- treated, supernatant- treated and MSCs + supernatant treated group. Each group (n = 7) was housed in individual standard cages at temperature of 20-25°C and 45-55% humidity under a 12/12 h light/dark cycle. All of the experiments were carried out in accordance with the guidelines of the Ethics Committee of Iran University of Medical Sciences.

### Isolation and cultivation of rat bone marrow-derived mesenchymal stem cells

Bone marrow (BM) was isolated from femurs and tibias of two-month-old male Wistar rats (200–250 g) under aseptic condition. Wistar rats were euthanized by ketamine (150 mg/kg, ip)- Xylazine (10 mg/kg, ip). Femur and Tibia were dissected and soaked in cold PBS. Adherent soft tissues was carefully removed. BM was exposed and aspirated by forcing 4–5 ml cell culture medium. BM cells (including hematopoietic stem cell and marrow stromal cells) were cultured in minimal essential medium α (α-MEM, Gibco, Invitrogen, Carlsbad, CA, USA) containing 15% fetal bovine serum (FBS, Gibco, Invitrogen, Carlsbad, CA, USA) and 1% penicillin/streptomycin (Gibco, Invitrogen, Carlsbad, CA, USA) and incubated at 37°C and 5% CO2. After 24 h, medium was changed and adherent cells were fed with fresh medium every 2–3 days. After 10–14 days, adherent cells formed homogenous fibroblast-like colonies. The cells reach to passages 3–5 were used for following studies. Flowcytometry analysis was used for approving of MSCs and their multipotent property.

### Flow cytometry analysis of MSCs

Cultured MSCs were detached by Trypsin/EDTA and washed twice with PBS. About 2× 10^5^ of cells were incubated with an appropriate concentrations of FITC-conjugated monoclonal rat antimouse CD11b, CD31, CD34, CD44, CD45, CD73, CD90, CD105 and CD106 for 40 min at 4°C in the dark. The cells were washed by centrifugation for 5 min and resuspended in PBS. Quantitative fluorescence analysis was carried out using FACS Calibur Cytometer (Becton Dickinson, San Diego, CA, USA) and Cell Quest software. At least 20 000 events were collected. All the experiments also incubated with FITC- rat anti-mouse IgG1 as a negative isotype control.

### STZ-induced diabetes in male Wistar rats

Diabetic groups received single intra peritoneal injection of 65 mg/kg Streptozocin (STZ, Sigma-Aldrich, St. Louis, MO, USA). The control group received sodium citrate buffer. STZ was dissolved in sodium citrate buffer, pH 4.5, and injected immediately after preparation. Blood glucose levels were monitored before and after injection of STZ by glucometer (GM300, Bionime Corporation, Taiwan). During the experience, from day 3 to 42, blood glucose of animals was measured weekly at 8:00–9:00 AM.

### Mesenchymsl stem cells transplantation

Animals were anesthetized by injection of a mixture of ketamine hydrochloride (100 mg/kg, ip) and xylazine (5 mg/kg, ip). The adherent MSCs were trypsinized and washed twice with PBS and 2.5 × 10^6^ cells/rat of MSCs in 1.0 ml saline were injected through tail vein. Second transplantation of MSCs was performed with interval of 20 days from the first transplantation. The same volume of normal saline was injected into diabetic control groups.



### Harvesting and administration of Supernatant of MSCs

MSCs of passage 3–5 were seeded for further expansion. After the medium of MSCs was changed once, conditioned medium of MSCs (supernatant) was collected at 48 hours and filtered through a 0.2-μm filter. Supernatant was preserved at -20°C until its following injection. Supernatant was injected daily (250 μl/rat, ip) into supernatant- treated group.

### Labeling of the MSCs for assessing of in-vivo migration

MSCs were labeled by fluorescence cell membrane tracker, CM-DiI™ (Invitrogen, Carlsbad, CA, USA). CM-DiI was prepared under manufacturer’s protocol. The medium of cultured cells was removed and 1 ml of CM -DiI was added to cells in T 25 cm^2^ flask. The cells was incubated in 37°C for 5 minutes and then in 4°C for 15 minutes. Then medium was added to cells and incubated for overnight. After 24 h, about 1 × 10^6^ MSCs was transplanted via tail vein injection. Twenty four hours after cell transplantation, animals were anesthetized and pancreases were obtained. The pancreas was then processed for paraffin embedding. Serial sections of tissues were prepared. The slides were deparaffinized and rehydration process was performed. Pancreatic tissues were then assessed under fluorescent microscope. The sections counterstained with Hoechst staining solution (Sigma-Aldrich, St. Louis, MO, USA).

### Blood glucose monitoring and insulin levels measurements

Blood glucose of all animals were measured weekly by glucometer for 42 days. Animals were considered diabetic when blood glucose levels were higher than 300 mg/dl for two consecutive times.

For serum insulin measurements, blood samples were collected from animals by weekly after intervention (week 1, 2, 4 and 6). Serum insulin levels were measured by rat insulin ELISA Kit (Mercodia AB Inc, Sweden) under the protocol of manufacturer.

### Immunohistochemical analysis

Animals were anesthetized and perfused intracardially with 4% paraformaldehyde/PBS. Pancreatic tissues were dissected and soaked in 4% paraformaldehyde. Fixed tissues were then processed for paraffin embedding with tissue processing. Pancreatic sections stained with Hematoxylin/Eosin. Immunohistochemical detection of insulin was performed on 4 μm sections using a standard chain polymer-conjugated technique as described previously on paraffin-embedded tissues of pancreas specimens [25]. Briefly, after deparaffinization, tissue sections were immersed in methanol containing 0.3% hydrogen peroxide to block endogenous peroxidase activity. Protein blocker was then used for blocking non-specific binding. Antigen was retrieved by autoclaving in citrate buffer (pH 6.0). The sections were incubated with primary Anti- insulin antibody (guinea pig polyclonal to insulin, ab7842, Abcam, Cambridge, UK) at 4°C overnight and then incubated with rabbit polyclonal secondary antibody (Rabbit polyclonal secondary antibody to guinea pig IgG - H&L (HRP), ab6771, Abcam, Cambridge, UK) for 1 hour at room temperature. The slides were washed and visualized using 3, 3-diaminobenzidine, (DAB; DAKO, Glostrup, Denmark). The sections were counterstained with heamtoxylin (Dako, Glostrup, Denmark). Finally dehydration process was performed and slides were observed under light microscope.

### Statistical analyses

Statistical analysis was carried out by Prism (Version 5, Graph Pad Software Inc). All the group values were expressed as means ± SEM. Differences among groups were determined by a one-way ANOVA. P < 0.05 was considered as significant.

## Results

### Isolation and characterization of cultured MSCs

**Figure 1:**
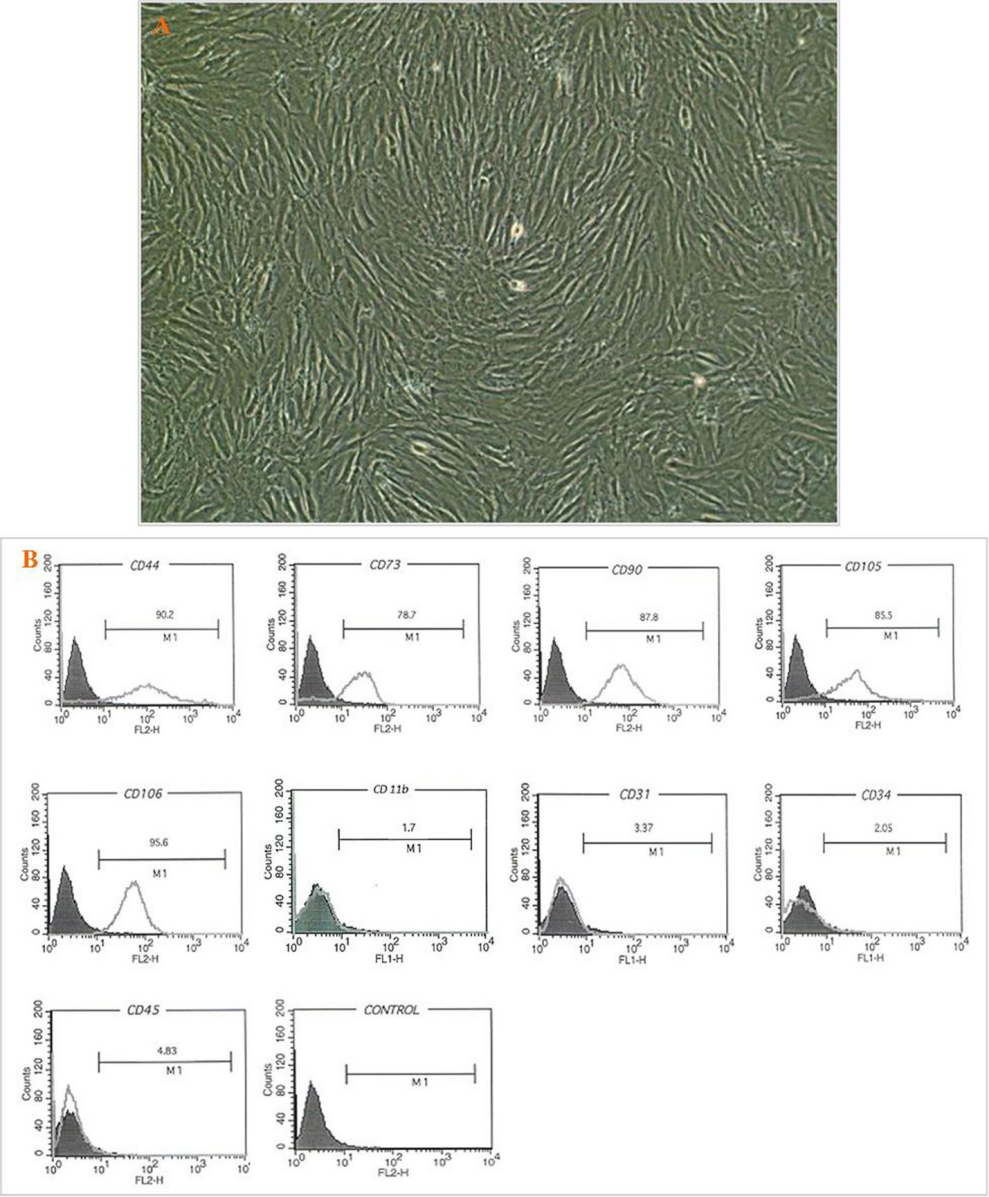
**Morphology of mesenchymal stem cells and their characterization. A**, Mesenchymsl stem cells in passage 3, cells with fibroblast- like morphology. **B**, Characterization of rat BM-MSC surface markers using flow cytometry. Flow cytometric analyses showed that cultured cells were positive for CD44, CD73, CD90, CD105 and CD106. These cells were negative for CD11b, CD31, CD34, and CD45. The respective isotype control is shown as black line. The data are representative of three independent experiments.

The isolated MSCs showed homogenous fibroblastic like morphology in vitro that tightly attached to the culture dish (Figure 1). Results of flow cytometric analysis on expression of cell surface antigens shown that MSCs were expressed CD44, CD73, CD105, CD106, and CD90 while were negative for CD11b, CD31, CD34, and CD45 (Figure 1).

### Tracking of transplanted MSCs in pancreatic tissues

**Figure 2:**
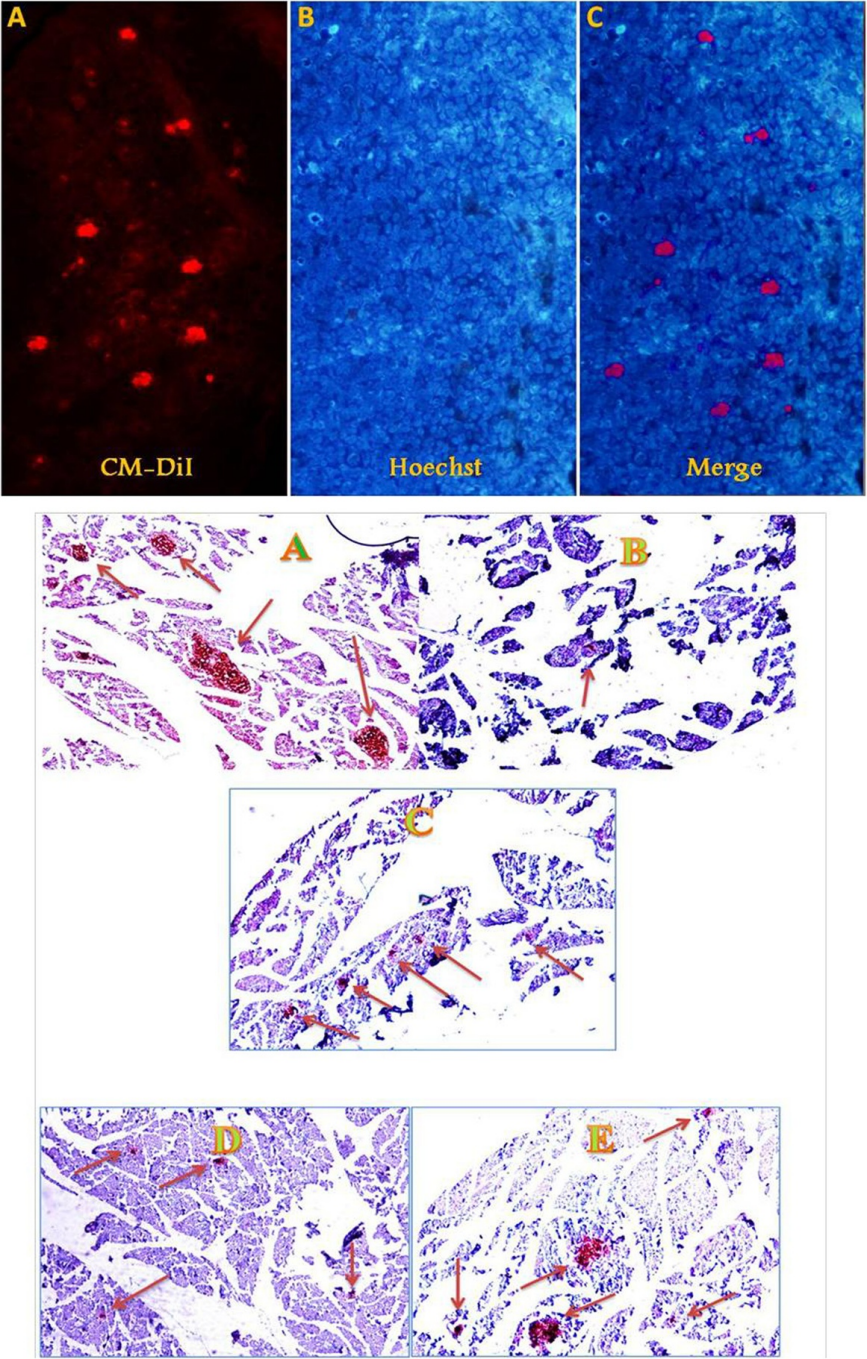
**I, Assessment of CM-Dil** ^**+**^ **-MSCs homing into pancreatic tissues 24 hours after transplantation. (A)** Image of MSCs implantation in the pancreas (red points). **(B)** Counterstaining staining was performed using Hoechst (Blue). **II**, Immunohistochemistry staining of pancreatic tissues of different animals with anti-insulin antibody at the end of study. Normal control **(A)**, diabetic control, islets were destroyed after exposing with STZ **(B)** MSCs-treated group **(C)**, supernatant treated group **(D)**, and MSCs + supernatant treated group **(E)** at the end of experiment. Size and number of islets were increased after the treatment particularly in MSCs + supernatant treated group. Arrows, islets of Langerhans. Sections (5 μm) are magnified × 200.

After transplantation of CM-DiI^+^-MSCs, serial sections of the pancreatic tissues were assessed to detect migration of CM-DiI -positive cells in pancreatic section. The sections stained with CM-DiI were fire red under fluorescent microscope (Figure 2).

### Induction of diabetes type 1

**Figure 3:**
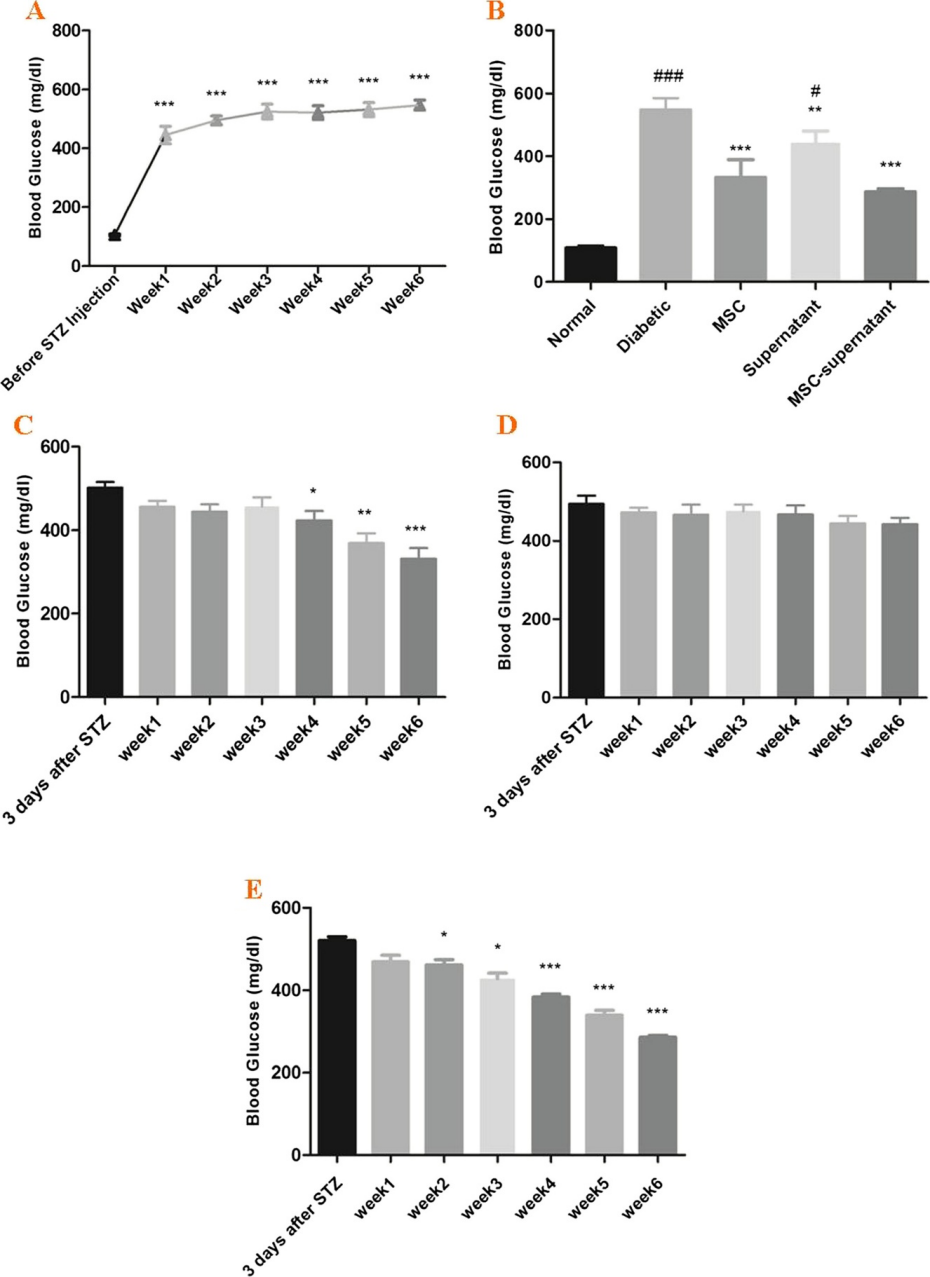
**Blood glucose levels in normal, diabetic and treated rats during the experiment.** All values are mean ± SEM. **A**, Blood glucose levels in diabetic controls during the weeks of experiment, elevated blood glucose levels were continued up to the end of experiment. *, blood glucose values at weeks 1–6 vs. 3 days after STZ. **B**, **C**, **D**, blood glucose levels in supernatant, MSCs and MSCs + supernatant -treated groups respectively during the weeks of experiment. *; blood glucose values at weeks 1–6 vs. 3 days after STZ. **E**, blood glucose values in different groups at the end of study. *, MSC, supernatant, MSC-supernatant groups vs. diabetic control. #, diabetic, supernatant, MSC-supernatant groups vs. MSC group. *,P < 0.05; **, P < 0.01; ***, P < 0.001. #, P < 0.05; ##, P < 0.01; ###, P < 0.001.

Three days after STZ injection, most rats became hyperglycemic with mean blood glucose levels of 445.40 ± 29.91. Elevated blood glucose levels were continued up to the end of experiment (547.20 ± 17.07) (Figure 3). Also, Immunohistochemical analysis of pancreatic tissues at the end of study showed that islets of pancreas were almost destroyed after receiving a single dose of STZ (Figure 2).

### Effect of MSCs transplantation, supernatant administration and co-administration of MSCs with supernatant on blood glucose of rats

The effect of transplantation of MSCs, supernatant and co-administration of MSCs with supernatant on blood glucose is shown in Figure 3. There was significant difference in blood glucose values among the weeks of experiments (1–6 weeks) in all treated group. Second transplantation of MSCs reduced blood glucose levels at week 4, while MSCs + supernatant administration significantly reduced blood glucose from week 2 after treatment (Figure 3). Results showed that supernatant of MSCs could not reduce blood glucose as good as MSCs. Blood glucose was significantly reduced in MSCs + supernatant treated group compared to MSCs and supernatant groups at the end of experiment (Figure 3).

### Effect of MSCs transplantation, supernatant administration and co-administration of MSCs with supernatant on insulin levels of rats

**Figure 4:**
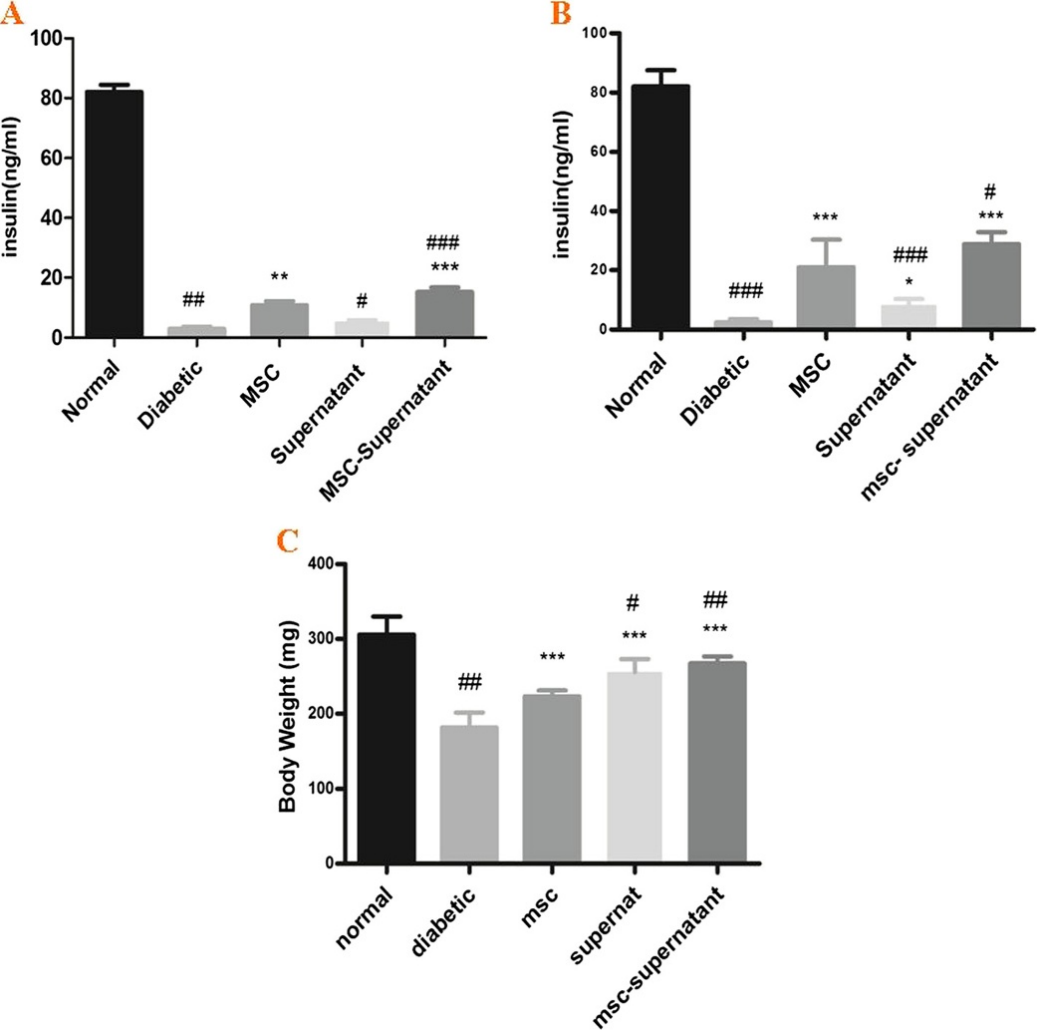
**Effect of MSCs transplantation, supernatant administration and co-administration of MSCs with supernatant on serum insulin levels and body weights in different animals.** Values are mean ± SEM. **A**, serum insulin levels at week 4. **B**, serum insulin levels at the end of study at week 6. **C**, body weight of animals at the end of study. *; MSC, supernatant, MSC-supernatant groups vs. diabetic control group. #; Diabetic, supernatant, MSC-supernatant groups vs. MSC group.*, P < 0.05; **, P < 0.01; ***,P < 0.001. #, P < 0.05; ##,P < 0.01; ###, P < 0.001.

MSC-treated group exhibited significant higher insulin levels after the second transplantation at week four, (10.80 ± 1.32 vs. 3.00 ± 1.58, P < 0.01) when compared to diabetic normal group. Also there was a significant increase in insulin levels in MSCs + supernatant group when compared to diabetic normal group (15.20 ± 1.530 vs. 3.00 ± 1.58, P < 0.001) at week four. The results showed that MSCs + Supernatant increased serum insulin levels more than MSCs at week four. Unlike, there was no significant difference between insulin levels values in supernatant treated group when compared to diabetic animals at week four (5.40 ± 1.14 vs. 3.00 ± 1.58) (Figure 4).

At the end of study, day 42, significant difference in insulin levels was observed in all treated groups; MSCs treated (21.00 ± 4.16 vs. 2.40 ± 1.14, P < 0.001), supernatant treated (8.12 ± 2.05 vs. 2.40 ± 1.14, P < 0.05) and MSCs + supernatant treated animals (28.80 ± 1.83 vs. 2.40 ± 1.14, P < 0.001) compared to diabetic controls. Also, our results indicated that MSCs + supernatant treated group showed higher increase in insulin levels when compared to MSC treated group (P < 0.05) (Figure 4).

### Immunohistochemical analysis of pancreatic tissues

Immunohistochemical assessment of pancreas indicated that both MSCs and supernatant initiates pancreatic regeneration after transplantation into recipient diabetic rats. Partial pancreatic renewal was evidenced by emerge of small islets in pancreatic tissues. The number and size of islets per section in MSCs treated animals were higher than supernatant groups. Also the number and sizes of islets per section in MSCs + supernatant group were higher than other treated groups (Figure 2).

### Effect of MSCs, supernatant administration and co-administration of MSCs with supernatant on body weight of rats

Six weeks after induction of DM, diabetic controls exhibited significant reduction in their body weight compared to before induction of diabetes (182.20 ± 8.60 vs. 231.60 ± 8.35, P < 0.001). There was significant difference between body weight of MSCs -treated diabetic rats and diabetic control animals (223.20 g ± 3.50 vs. 182.20 g ± 8.60, P < 0.001). There was a significant increase in body weight of supernatant- treated rats when compared to diabetic control rats at week six (255.80 g ± 7.73 vs. 182.20 g ± 8.60, P < 0.01) (Figure 2). Our results also showed that body weight improvement in MSCs + supernatant received animals was significantly higher than MSCs and supernatant groups respectively (267.20 g ± 4.16 vs. 223.20 g ± 3.50, P < 0.01) (267.20 g ± 4.16 vs. 255.80 g ± 7.73, P < 0.05).

## Discussion

Mesenchymal stromal cells are multipotent stem cells showing tremendous potential for regenerative medicine due to their ability in: self-renewing, differentiation into a variety of tissues and their availability in almost all postnatal organs and tissues [26–28]. It has been proposed that the ability of MSCs to secrete a variety of factors that change tissue microenvironment functionally is more important than their trans-differentiation ability in affecting tissue regeneration [29, 30]. Therefore, besides their ability to differentiate into many cell lines, the secretion of a wide range of biological molecules by MSCs, such as growth factors, cytokines and chemokines, establishes their biologically effective role under condition of injury [29–33]. Although the exact mechanism of action of MSCs in tissue regeneration is poorly understood, MSCs therapy has attracted a great deal of attention for the treatment of diabetes mellitus. In this study we planned a comparative study on the influence of MSCs transplantation, supernatant administration and co-administration of MSCs with supernatant on control of hyperglycemia in STZ-induced diabetic rats.

In present study, migration of MSCs to injured tissues was demonstrated by observation of CM-DiI^+^-MSCs in pancreatic sections under fluorescent microscope. Small islets regeneration observed in immune-histochemical analysis accompanying with elevation of insulin levels and decrease in blood glucose levels in diabetic-MSCs-treated animals, indicating survival of transplanted MSCs in animal’s tissues.

Our results showed that, although single transplantation of MSCs could prevent further rise of blood glucose in diabetic rats, but it could not significantly control hyperglycemia as good as when repeated injection of MSCs were used. We found that single transplantation of 2.5 × 10^6^ MSCs could not reduce blood glucose levels of diabetic animals, but repeated injection of same dose of MSCs at the week of four, could significantly reduce blood glucose levels compared to diabetic controls. It is supposed that the first injection of MSCs provide a suitable environment by releasing some growth factor and cytokines, in which survival, docking and homing of incoming MSCs is being facilitated.

In contrary with our study, some evidences reported that single transplantation of MSCs could reduce blood glucose in STZ- induced diabetic rats [14, 34]. In the study designed by Dong and et.al, performing an allogeneic diabetic mesenchymal stem cells transplantation in STZ-induced diabetic rats, they reported that single injection of 2- 4 × 10^6^ MSCs reduced blood glucose levels in recipient rats compared to diabetic controls (27.8 ± 2.1 mM/l vs. 17.7 ± 3.9 mM/l , *P* < 0.05). They have indicated that small quantity of transplanted MSCs survived and transdifferentiated into insulin-producing cells in pancreas of recipient rats [14].

Efficacy and safety of repeated transplantation of MSCs have been assessed by some other studies in different organs. They exhibited that repeated administration of MSCs is more effective than single administration in improving damaged tissues [35–37]. Repeated versus single transplantation of MSCs for improving of liver injury in mice was studied by Miryounesi et al., they found that transplanting 3 × 10^6^ MSCs in three divided doses improved survival, liver fibrosis and necrosis compared to injection of the same number of MSCs in a single dose. They have also exhibited that repeated injection of MSCs was three times more effective in homing of transplanted cells compared to the single transplant [35].

Using supernatant of MSCs, so-called conditioned medium (CM), in tissue regeneration comes from the idea that MSCs could have beneficial effect even if they do not engraft in target tissues. Bi et al. demonstrated that administration of conditioned medium from bone marrow-derived MSCs in a model of acute kidney injury (AKI) increased survival and limited renal injury in mice. They have also showed that conditioned medium of MSCs, induced migration and proliferation of kidney-derived epithelial cells and significantly diminished cisplatin-induced proximal tubule cell death in vitro [38].

In the study by Timmers et al., conditioned medium of MSCs was used to improve myocardial infarction (MI) in pigs. They have found intravenous human MSCs-conditioned medium increased capillary density and preserved cardiac function through enhancing myocardial perfusion in pigs with MI [39].

Our finding indicated that although there was a difference between blood glucose and insulin levels of supernatant treated group compared to diabetic controls at the end of study but, it could not reduce blood glucose as efficient as MSCs .Interestingly our results showed that supernatant of MSCs could be more beneficial if it would be administered accompany with MSCs. Co-administration of MSCs with supernatant of MSCs in diabetic rats could significantly increase insulin (28.80 ± 4.09) and reduce blood glucose (285.8 ± 10.11) levels in experimental animals compared to other treated groups. We also found that co-administration of MSCs with supernatant reduced blood glucose from week 2 after treatment while, transplantation of MSCs reduced blood glucose levels at week 4. We suppose that supernatant could help MSCs to migrate toward damaged pancreatic tissues via providing a microenvironment for better homing.

Immunohistochemical analysis of pancreas showed an increase in size and number of islets in each slice in MSCs + Supernatant group compared to other treated group. Our results showed that co- administration of MSCs with supernatant could be an effective tool in regeneration of the injured pancreatic tissues. It seems that conditioned medium of MSCs which containing a variety of growth factors, cytokines and immunomodulatory factors, could help MSCs in better survival, proliferation and eventually homing toward damaged pancreatic tissues and higher differentiation into pancreatic cells then after.

We also found that diabetic control animals lost their body weight and became slim during the experiment. In contrast, administration of MSCs and their supernatant to diabetic animals not only could prevent loosing but also could increase their body weight significantly compared to normal diabetic rats. Co-administration of MSCs with supernatant have synergistic effect on the body weight of animals improving their body weight significantly more than either MSCs or supernatant treated animals.

## Conclusion

In summary, our study was designed to compare differentiation and paracrine properties of MSCs in control of diabetes mellitus induced by STZ. Our results indicated that co-administration of MSCs and supernatant could control the hyperglycemia in diabetic rats more than either group alone. Therefore, it could be assumed that there is synergistic effect between MSCs and supernatant of MSCs in pancreatic regeneration of diabetic animals. This approach may be considered to be applied in cell therapy of diabetic patients.

## Abbreviations

MSCs: Mesenchymal stem cells
IPCs: Insulin producing cells
T1DM: Type 1 diabetes mellitus
DM: Diabetes mellitus
STZ: Streptozocin.
